# Supplementary material for: Forecasting emergency department visits in the reference hospital of the Balearic Islands: The role of tourist and weather data
Source: PLoS One. 2026 Mar 13;21(3):e0343713. doi: 10.1371/journal.pone.0343713 (PMC12987453; doi:10.1371/journal.pone.0343713)
Supplement: S1 Text — (PDF) [file pone.0343713.s001.pdf]

S1 Original data columns

- *Año* (year of the visit)
- *Servicio alta* (service of registration. Most of the entries are *urgencias*)
- *Sexo* (sex of the patient at the time of the visit))
- *Edad* (age of the patient at the time of the visit)
- *Fecha y hora inicio urg* (date and time of entry at the ED)
- *Fecha y Hora Fin Urg* (date and time of exit from the ED.)
- *Tipo paciente* (if the patient is pediatric or not)
- *Procedencia* (why the patient attended the ED, i.e. their own decision, GP's decision, from another hospital...)
- *Motivo alta* (reason for discharge, i.e. hospitalized, dismissed, dead, run away...)
- *Triatge data hm* (time of triage, when applicable)
- *Nacionalidad*: (nationality of the patient)
- *Provincia residencia* (province of residence of the patient if they are a Spanish resident)
- *Pais residencia* (country of residence of the patient)
- *Centro salud* (health center associated with the patient)
