# Supplementary figures and images for: Forecasting emergency department visits in the reference hospital of the Balearic Islands: The role of tourist and weather data
Source: PLoS One. 2026 Mar 13;21(3):e0343713. doi: 10.1371/journal.pone.0343713 (PMC12987453; doi:10.1371/journal.pone.0343713)

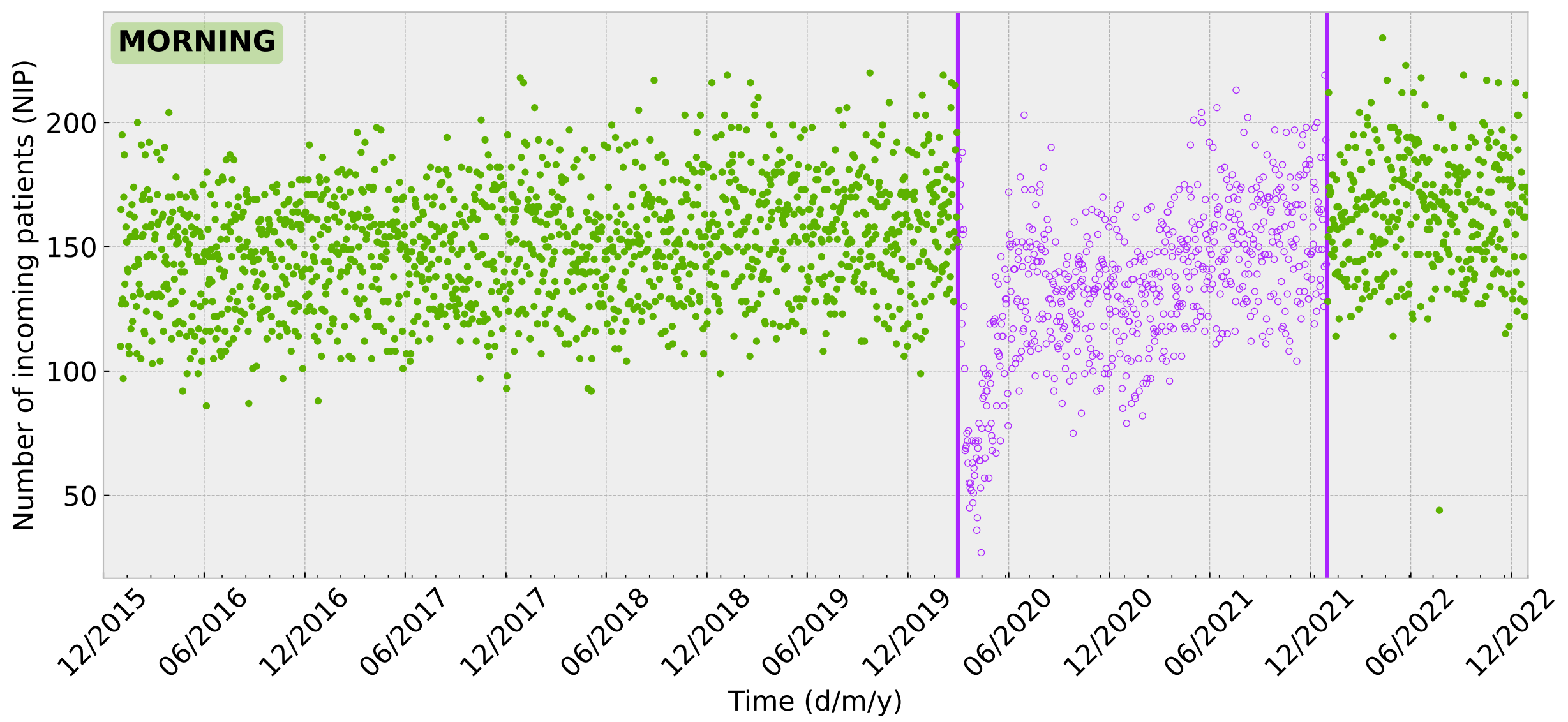

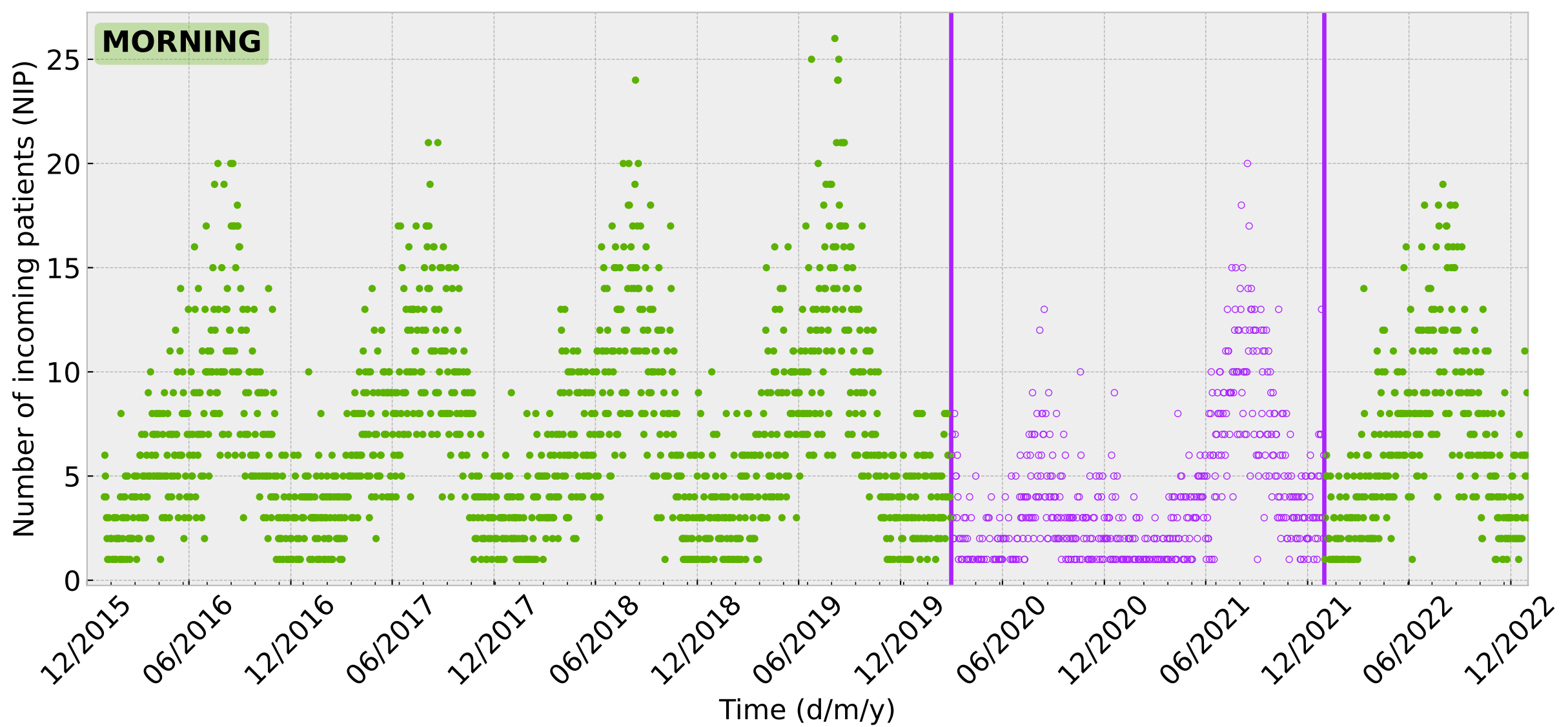

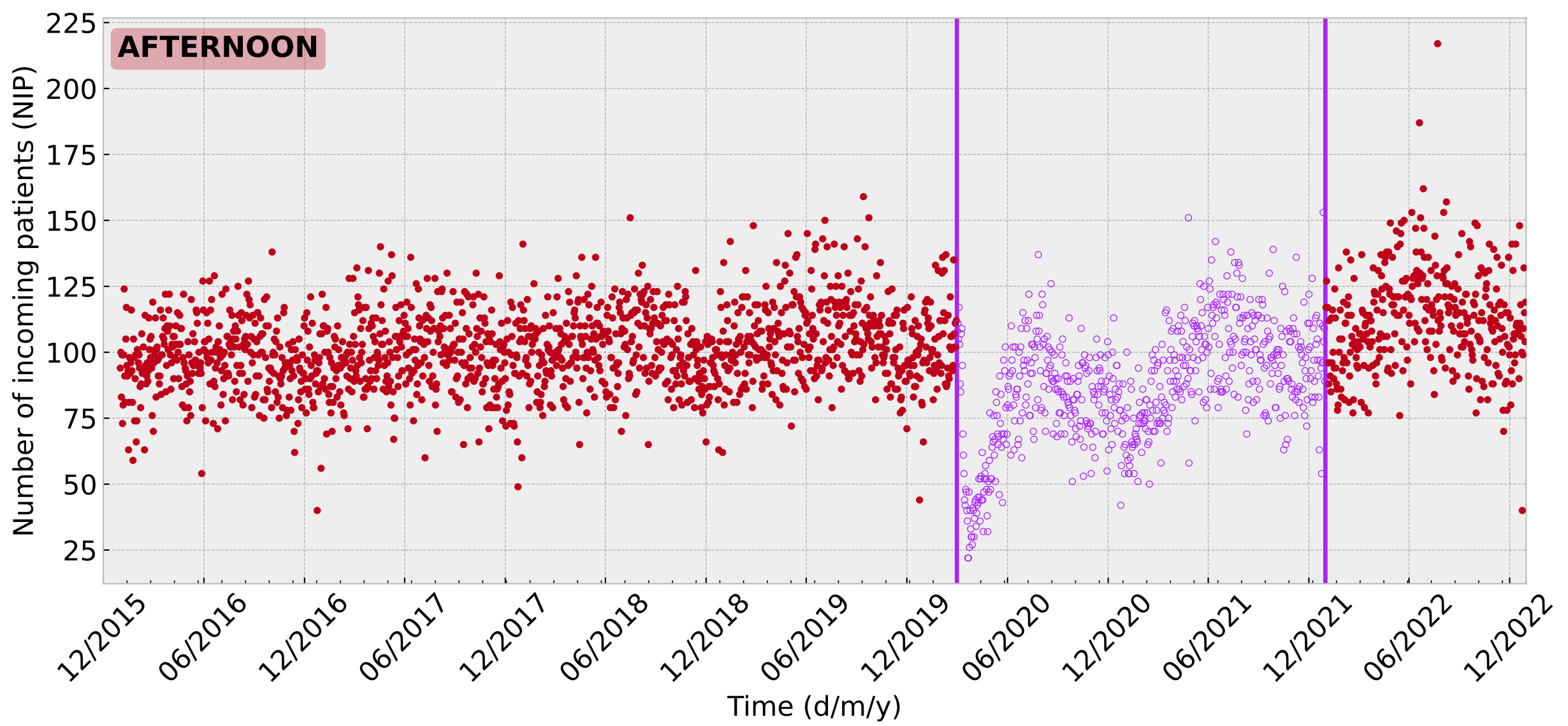

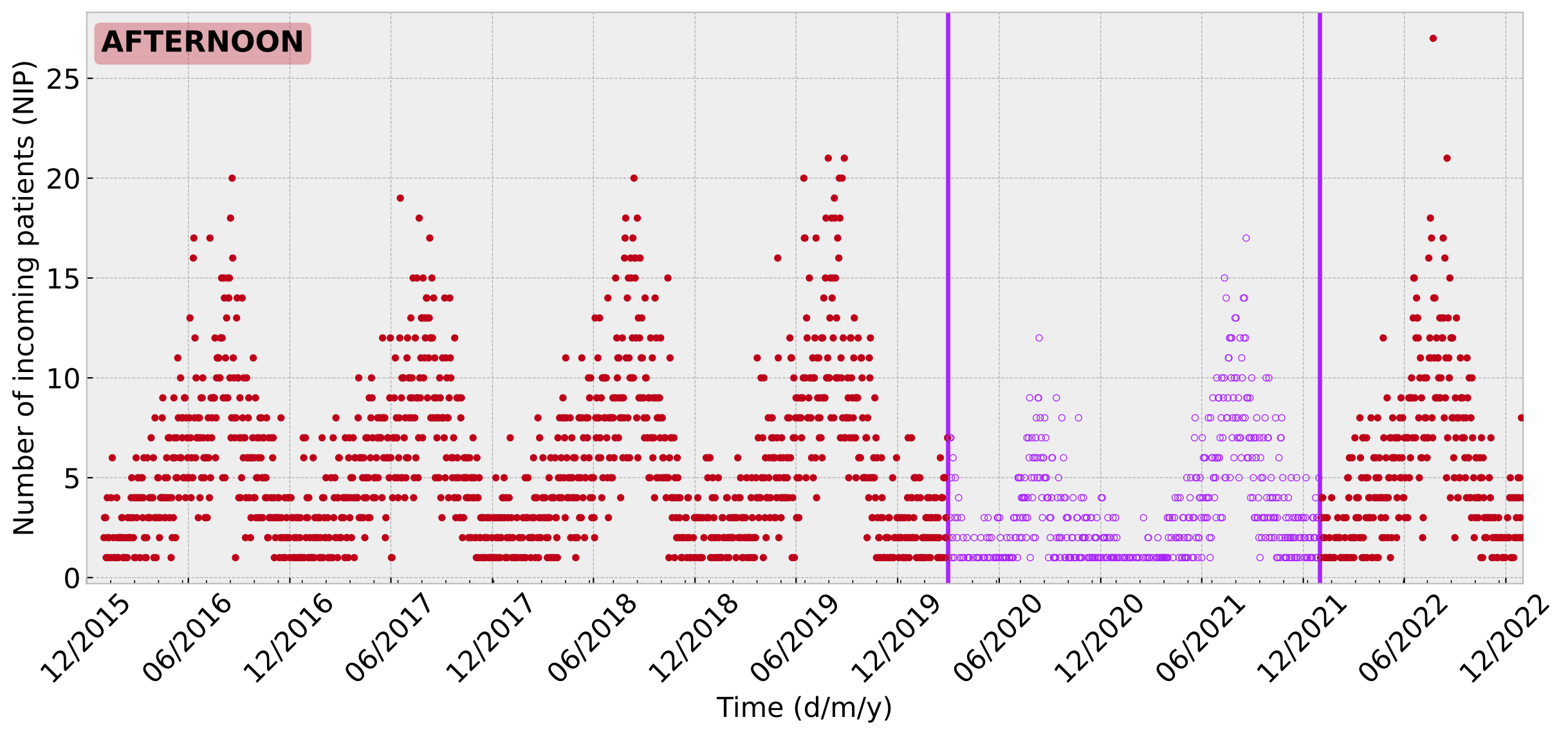

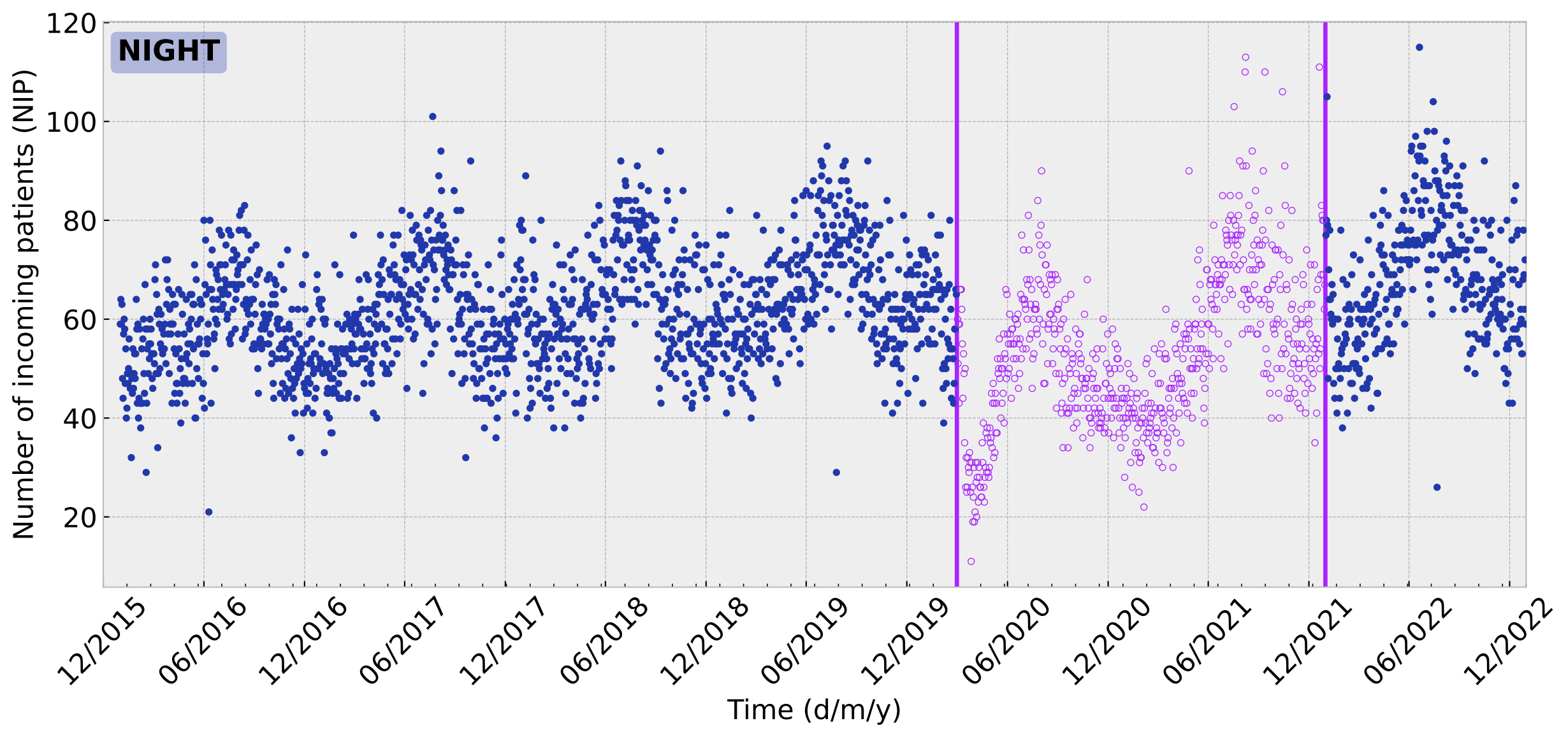

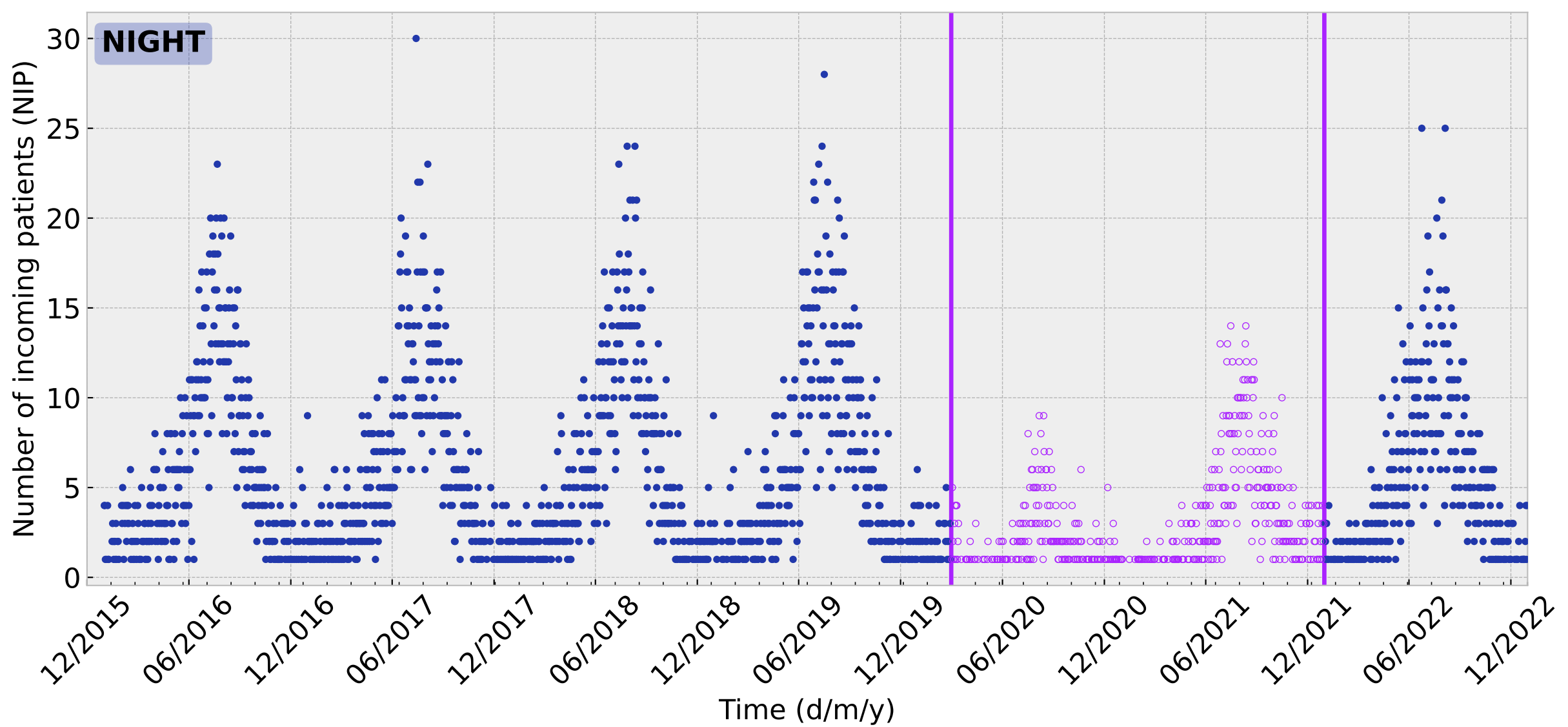

Supplement: S2 Fig — Subfigures on the left show the NIP for residents, and subfigures on the right show the NIP for non-residents. Each point corresponds to the NIP for a specific day and shift. Each subfigure shows a different shift (morning in green, afternoon in red, night in blue). The purple points between the dates March 1, 2020 and December 31, 2021 are the values registered during the assumed pandemic period, and excluded from our analysis. (PDF) [file pone.0343713.s002.pdf]

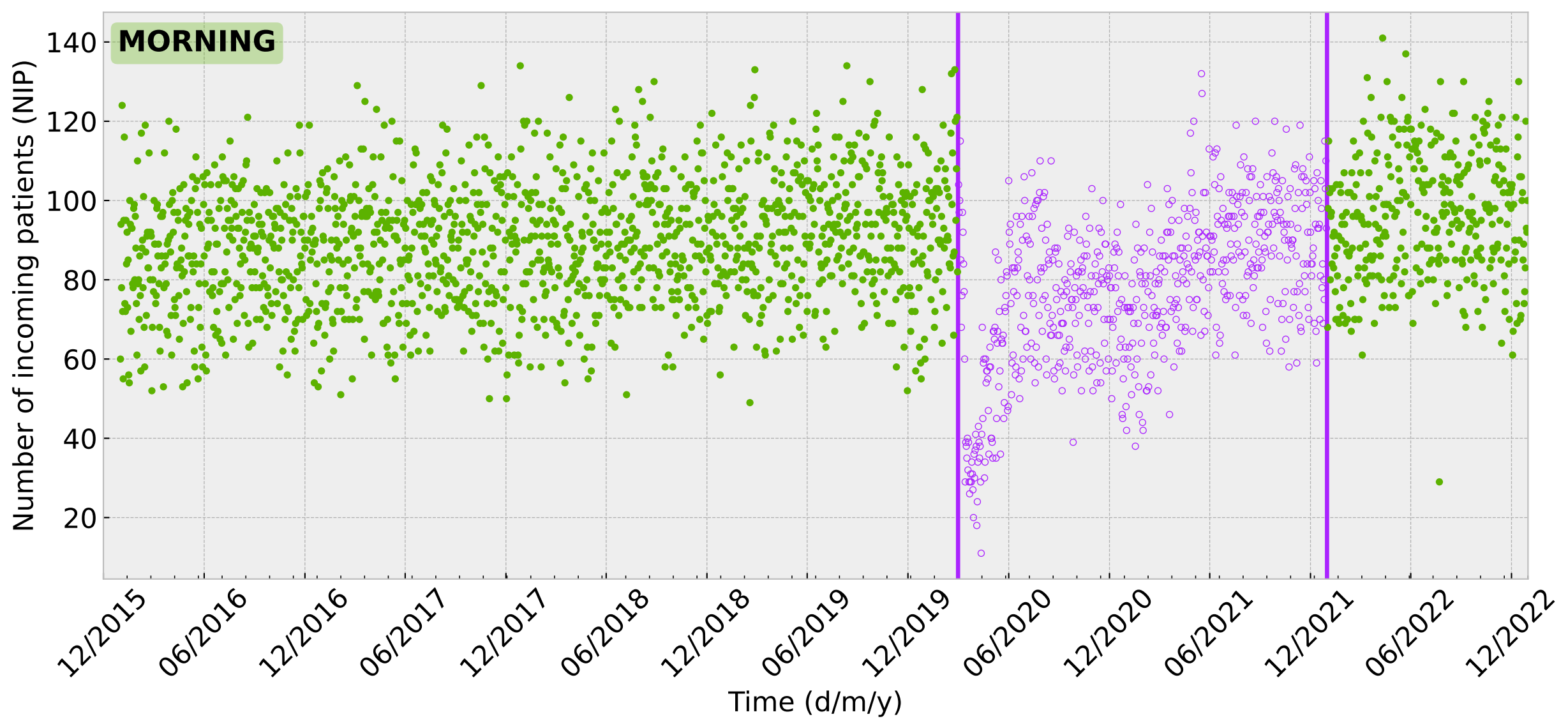

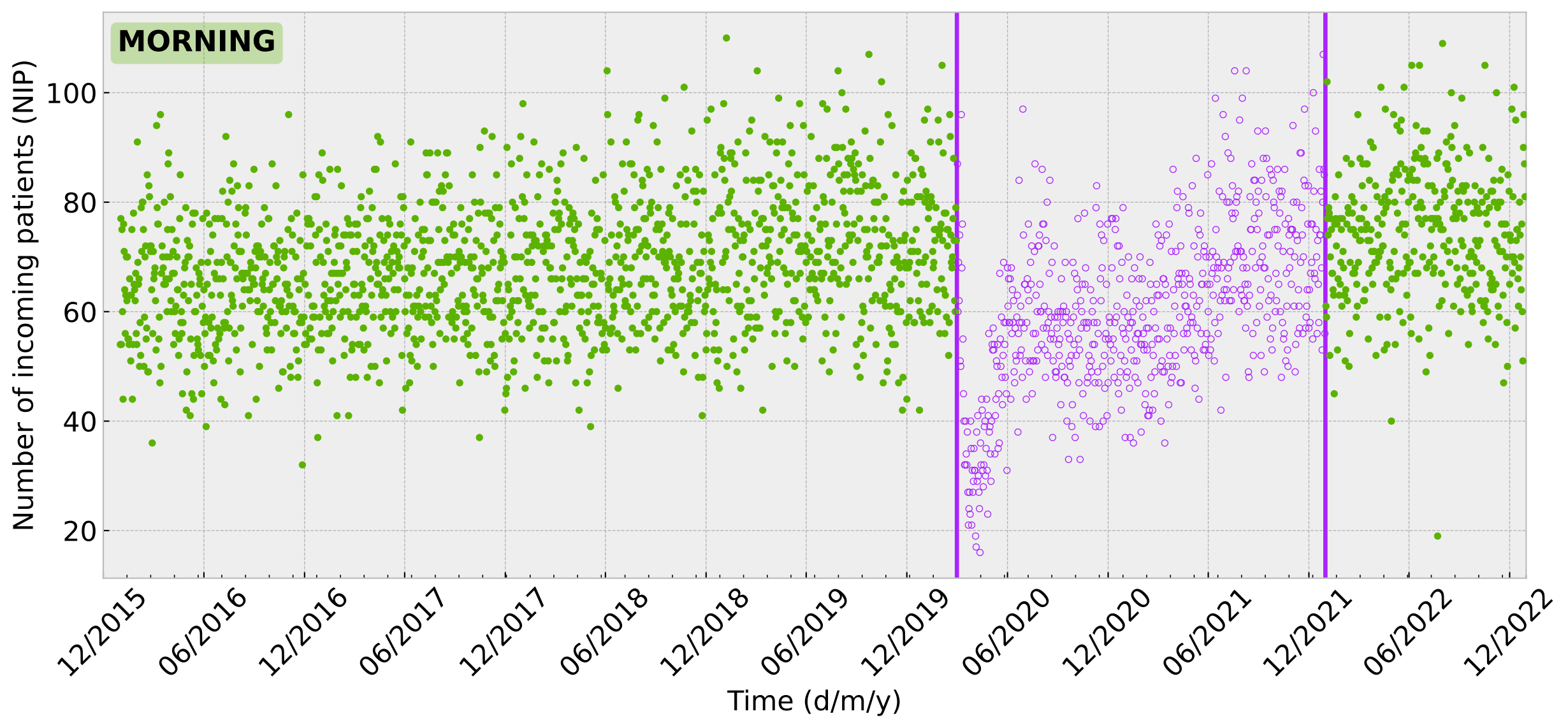

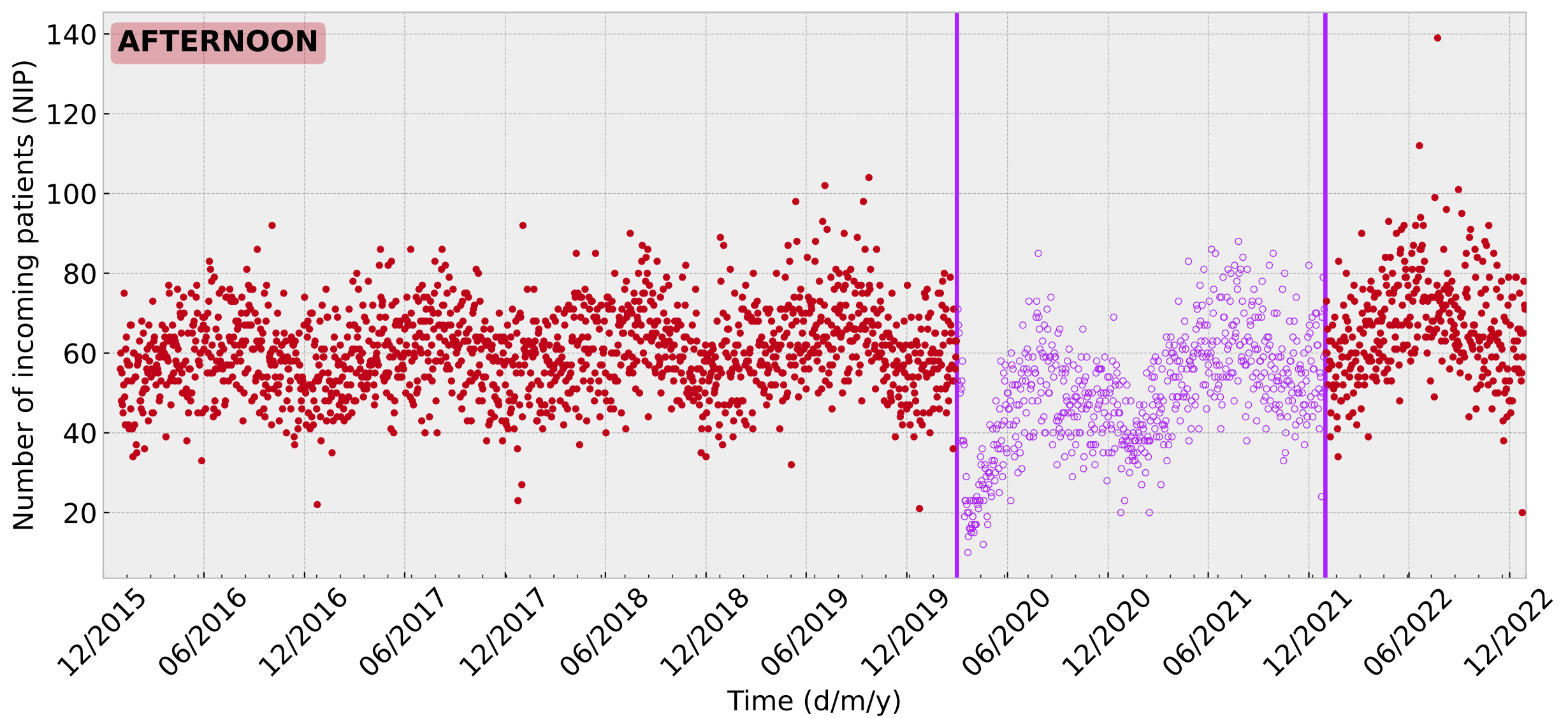

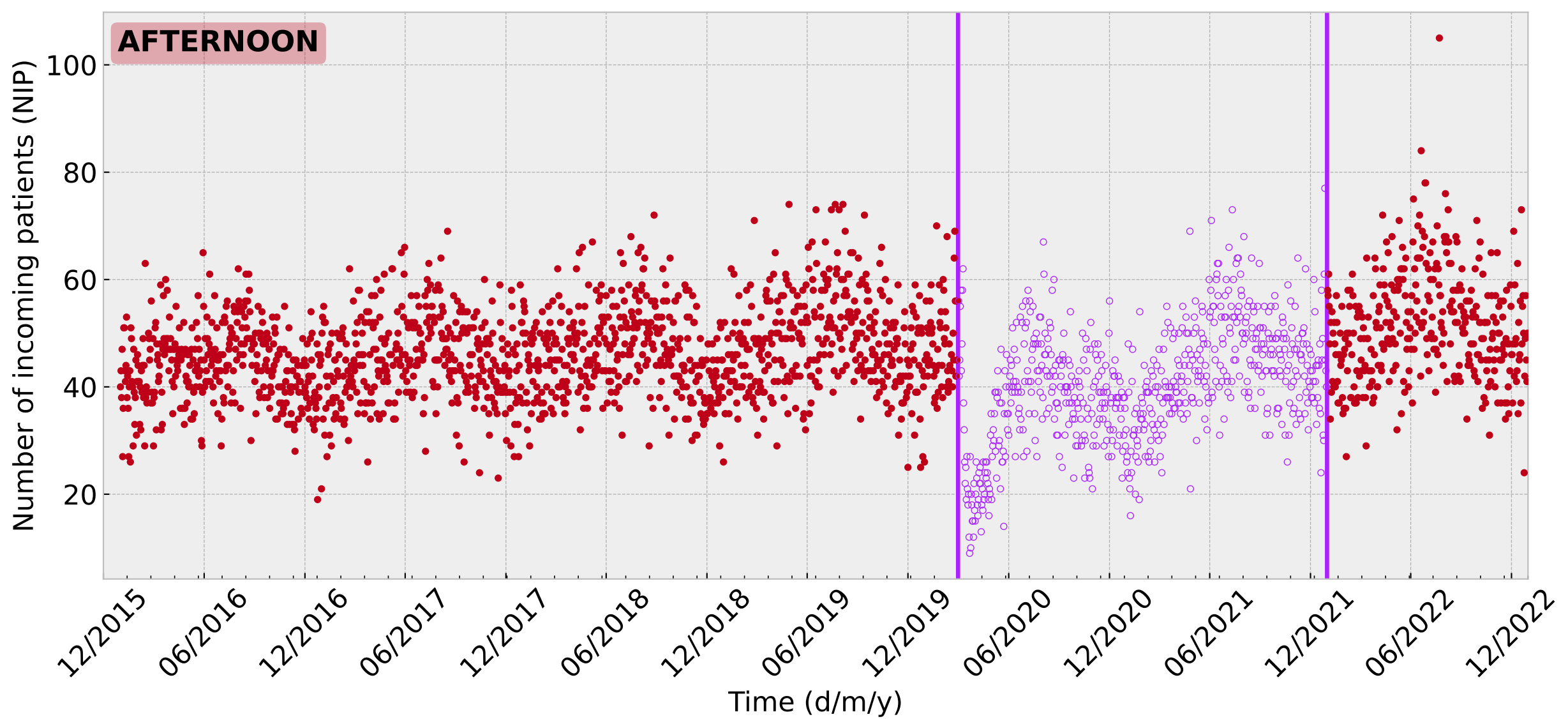

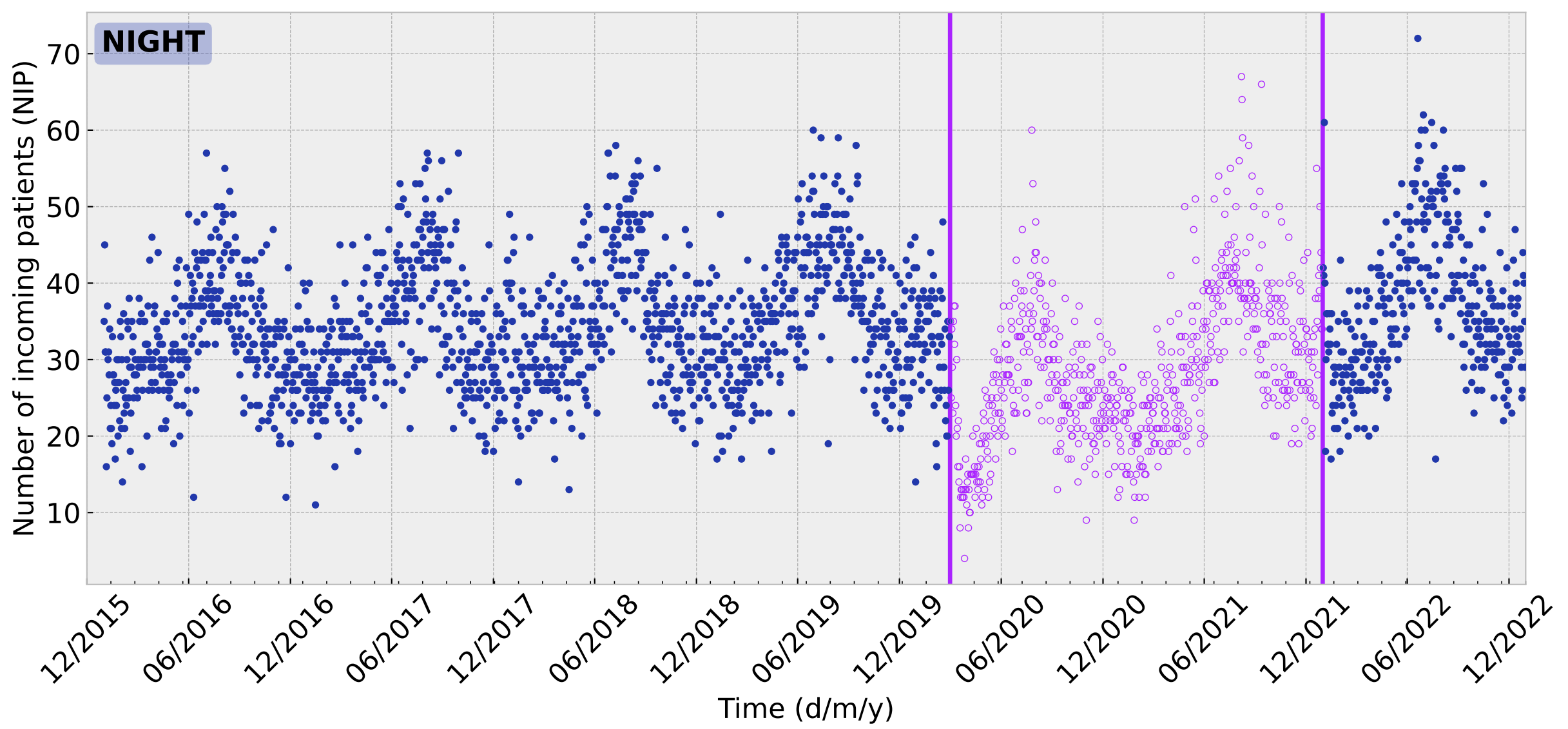

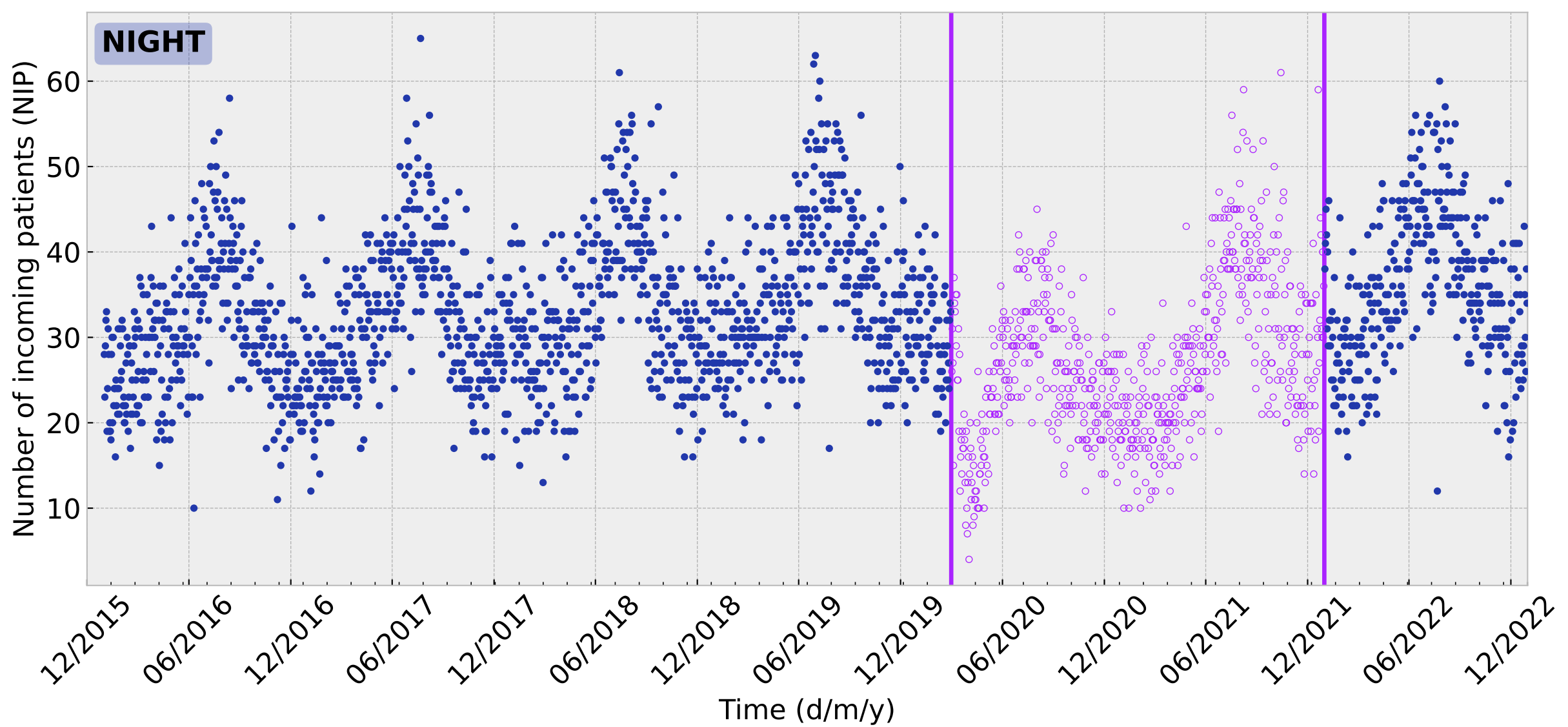

Supplement: S3 Fig — Subfigures on the left show the NIP for female patients, while subfigures on the right show the NIP for male patients. Each point corresponds to the NIP for a specific day and shift. Each subfigure shows a different shift (morning in green, afternoon in red, night in blue). The purple points between the dates March 1, 2020 and December 31, 2021 are the values registered during the pandemic, and excluded from our analysis. The behaviour of the curves on the left and right respectively are very similar to one another, thus eliminating the need to develop a different model for each sex. (PDF) [file pone.0343713.s003.pdf]

Random Forest

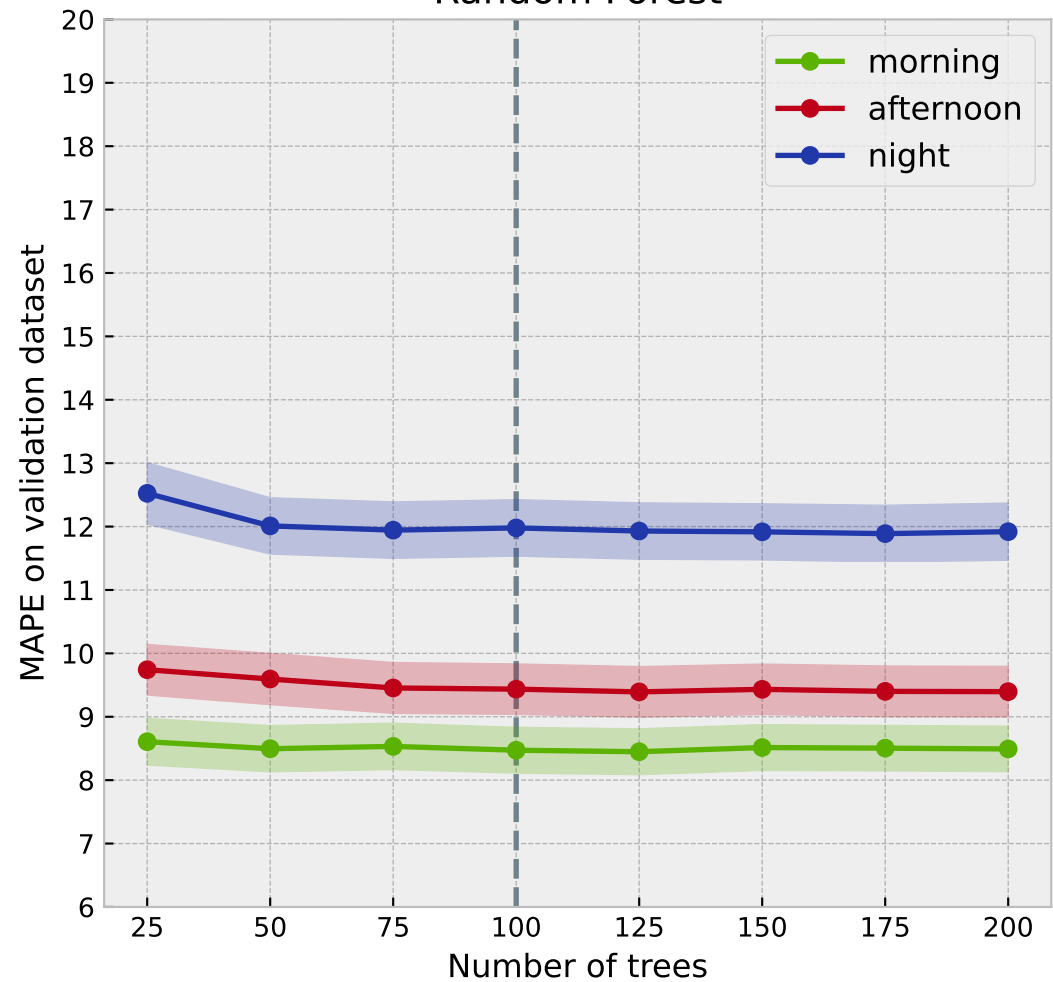

Support Vector Regressor

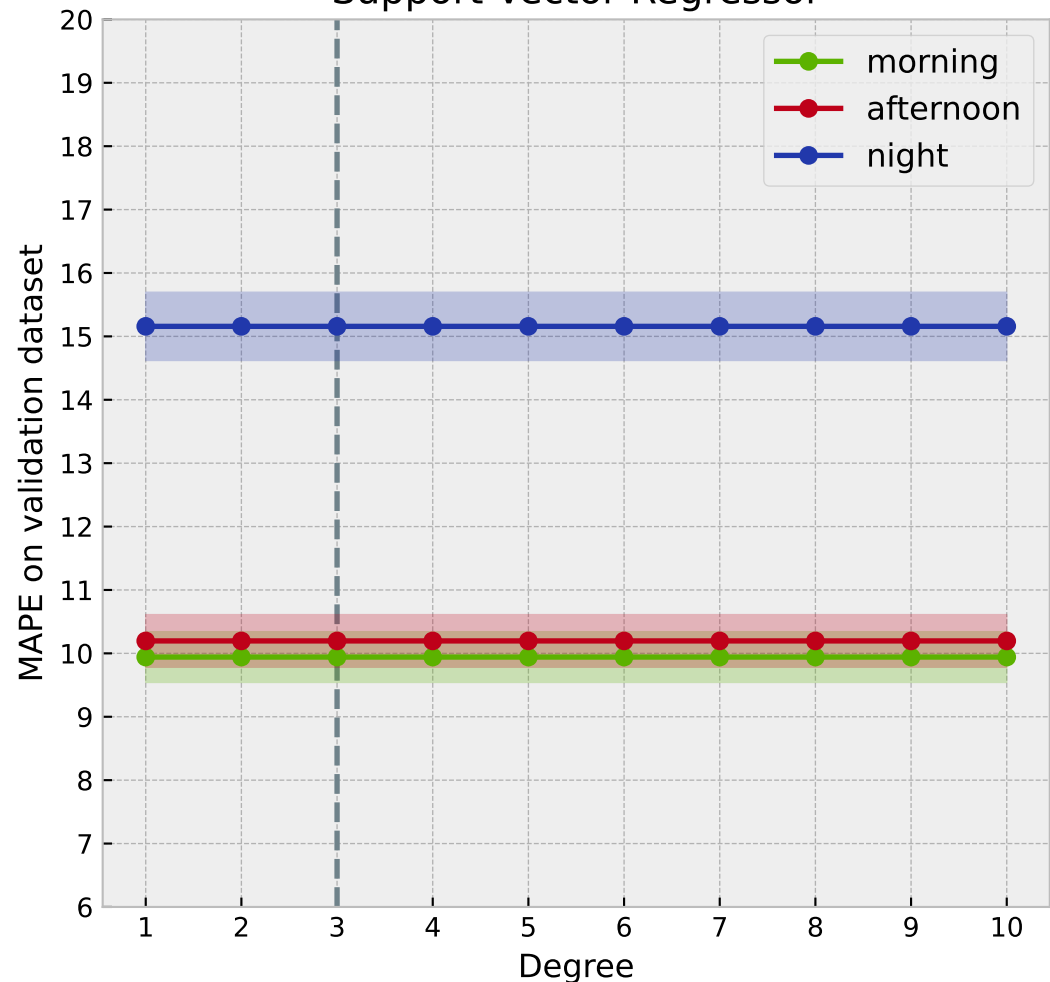

Supplement: S5 Fig — Subfigure on the left shows the SMAPE for the validation dataset obtained from the RF model for different values of the n_estimators hyperparameter. Subfigure on the right shows the SMAPE for the validation dataset obtained from the SVR model for different values of the degree hyperparameter. The vertical grey lines indicate the optimal values. (PDF) [file pone.0343713.s005.pdf]

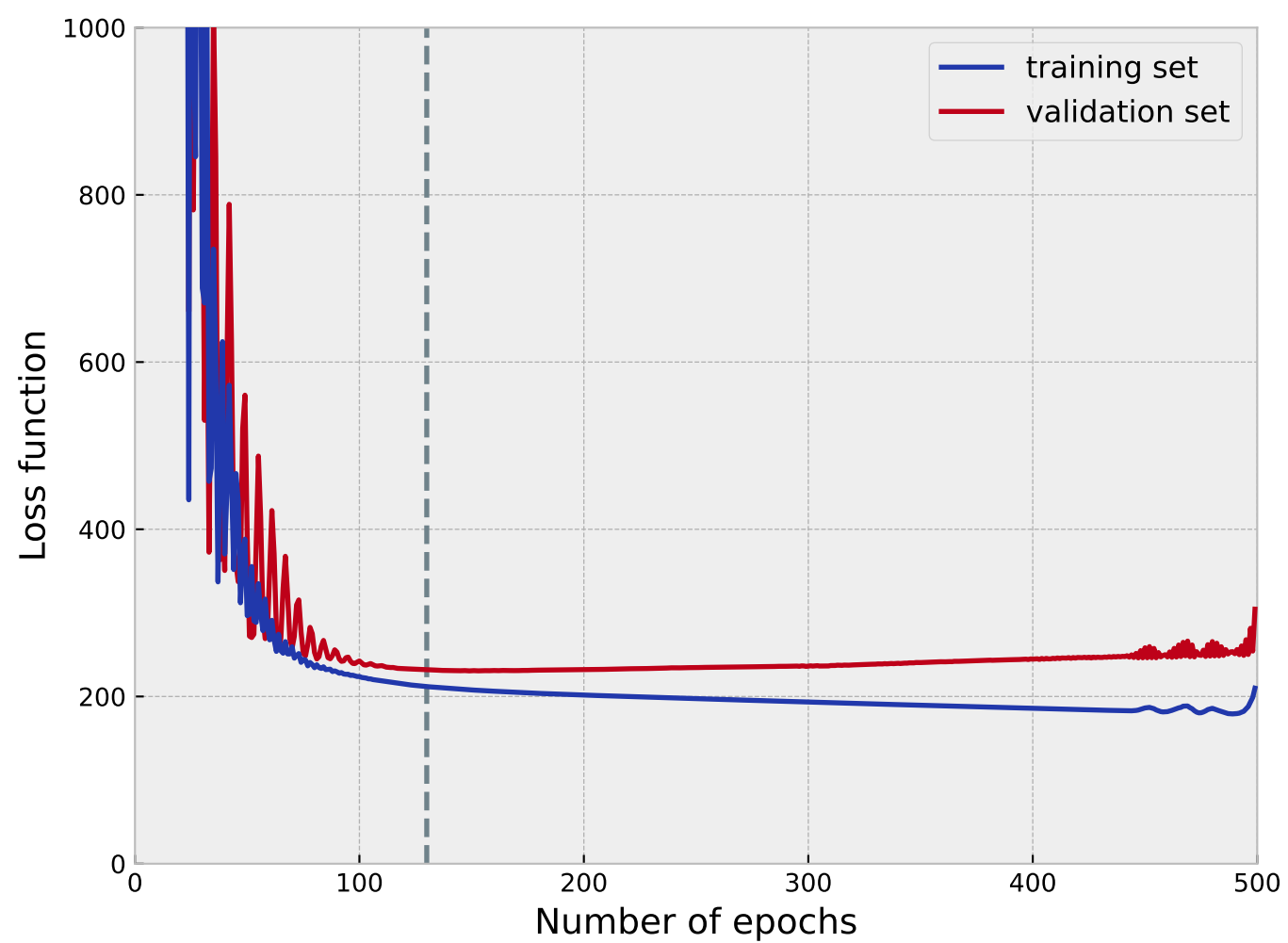

Supplement: S7 Fig — The figure shows the loss function at different training epochs for both the training (blue curve) and validation (red curve) dataset. The dashed vertical grey line indicates the optimal value, corresponding to the minimum of the validation curve. (PDF) [file pone.0343713.s007.pdf]
