# Supplementary material for: Forecasting emergency department visits in the reference hospital of the Balearic Islands: The role of tourist and weather data
Source: PLoS One. 2026 Mar 13;21(3):e0343713. doi: 10.1371/journal.pone.0343713 (PMC12987453; doi:10.1371/journal.pone.0343713)
Supplement: S4 Text — (PDF) [file pone.0343713.s004.pdf]

S4 DeepSeek prompt to preprocess weather data

You need to extract from the following spanish text information about the wind speed and the precipitation probability. You have to encode it as integer numbers from 0 to 4 as follows:

0 = none
1 = low
2 = medium
3 = high
4 = very high

Reply just with two numbers separated by a single space (nothing else in your output message!) where the first one is the precipitation probability and the second one is the wind speed that you infer from the text, both of them from 0 to 4 as described above.
