# Supplementary material for: Forecasting emergency department visits in the reference hospital of the Balearic Islands: The role of tourist and weather data
Source: PLoS One. 2026 Mar 13;21(3):e0343713. doi: 10.1371/journal.pone.0343713 (PMC12987453; doi:10.1371/journal.pone.0343713)
Supplement: S6 Text — Here nn corresponds to the module torch.nn. The main hyperparameter is the number of training epochs. To tune this hyperparameter, we ran the model for 500 epochs and checked the loss function of both training and validation sets at each epoch. In order to minimize overfitting, we set the number of epochs to 130 (see S7 Fig. Hyperparameter tuning for the FNN model). (PDF) [file pone.0343713.s006.pdf]

S6 FNN detailed structure

```
FNN = nn.Sequential(nn.Linear(X.shape[1], 32), nn.ReLU(),
                    nn.Linear(32, 64), nn.ReLU(),
                    nn.Linear(64, 128), nn.ReLU(),
                    nn.Linear(128, 256), nn.ReLU(),
                    nn.Linear(256, 512), nn.ReLU(),
                    nn.Linear(512, 1024), nn.ReLU(),
                    nn.Linear(1024, 2048), nn.ReLU(),
                    nn.Linear(2048, 1024), nn.ReLU(),
                    nn.Linear(1024, 512), nn.ReLU(),
                    nn.Linear(512, 256), nn.ReLU(),
                    nn.Linear(256, 128), nn.ReLU(),
                    nn.Linear(128, 64), nn.ReLU(),
                    nn.Linear(64, 32), nn.ReLU(),
                    nn.Linear(32, Y.shape[1])
                    )
criterion = nn.MSELoss()
optimizer = torch.optim.Adam(FNN.parameters(), lr=0.01)
```
