# Supplementary material for: Forecasting emergency department visits in the reference hospital of the Balearic Islands: The role of tourist and weather data
Source: PLoS One. 2026 Mar 13;21(3):e0343713. doi: 10.1371/journal.pone.0343713 (PMC12987453; doi:10.1371/journal.pone.0343713)
Supplement: S8 Table — Performance metrics (SMAPE, RMSE, MAE) and associated standard deviations across models and input variables. The metrics have been averaged across 1000 bootstrap samples. First table: shift-based predictions. Second table: risk-group-based predictions. (PDF) [file pone.0343713.s008.pdf]

Method	Input variable	Shift	SMAPE (mean)	SMAPE (st.dev.)	RMSE (mean)	RMSE (st.dev.)	MAE (mean)	MAE (st.dev.)
SARIMA-1		Morning	7.69	0.36	16.44	0.79	12.46	0.56
SARIMA-1		Afternoon	9.14	0.41	13.24	0.61	10.16	0.43
SARIMA-1		Night	10.48	0.48	9.70	0.57	7.47	0.33
SARIMAX-1		Morning	8.16	0.37	17.25	0.80	13.32	0.59
SARIMAX-1		Afternoon	10.99	0.47	15.54	0.67	12.15	0.49
SARIMAX-1		Night	12.23	0.51	10.91	0.49	8.66	0.35
SARIMA-7		Morning	7.84	0.37	16.74	0.78	12.73	0.57
SARIMA-7		Afternoon	9.47	0.41	13.58	0.59	10.48	0.44
SARIMA-7		Night	11.39	0.51	10.44	0.56	8.12	0.35
SARIMAX-7		Morning	12.02	0.57	29.79	2.60	20.49	1.11
SARIMAX-7		Afternoon	17.16	0.94	34.01	3.44	20.36	1.40
SARIMAX-7		Night	19.28	0.90	22.67	2.98	14.21	0.93
SARIMA-14		Morning	8.12	0.37	17.16	0.77	13.18	0.58
SARIMA-14		Afternoon	9.74	0.41	13.73	0.58	10.76	0.44
SARIMA-14		Night	12.28	0.51	11.10	0.57	8.79	0.36
SARIMAX-14		Morning	17.23	0.90	55.97	9.10	31.30	2.48
SARIMAX-14		Afternoon	26.66	1.75	75.35	9.76	35.42	3.53
SARIMAX-14		Night	31.02	1.49	44.66	6.52	23.64	1.99
SARIMA-28		Morning	8.52	0.38	17.90	0.76	13.78	0.60
SARIMA-28		Afternoon	10.48	0.45	14.95	0.58	11.61	0.48
SARIMA-28		Night	13.58	0.59	12.73	0.61	9.83	0.43
SARIMAX-28		Morning	30.22	1.56	109.52	19.23	55.46	5.11
SARIMAX-28		Afternoon	50.23	2.81	174.03	21.72	78.49	8.19
SARIMAX-28		Night	63.86	3.07	95.48	12.80	48.09	4.23
RF	All	Morning	9.21	0.36	18.65	0.71	14.92	0.60
RF	All	Afternoon	10.02	0.44	14.77	0.70	11.08	0.49
RF	All	Night	11.73	0.50	10.88	0.51	8.27	0.36
RF	No W	Morning	9.03	0.36	18.33	0.71	14.58	0.60
RF	No W	Afternoon	10.29	0.45	15.03	0.71	11.35	0.50
RF	No W	Night	12.08	0.49	11.07	0.49	8.54	0.36
RF	No T	Morning	9.48	0.36	18.97	0.70	15.28	0.60
RF	No T	Afternoon	10.79	0.47	15.67	0.72	11.86	0.53
RF	No T	Night	13.65	0.55	12.38	0.53	9.57	0.40
RF	No W-No T	Morning	9.43	0.36	18.99	0.71	15.28	0.59
RF	No W-No T	Afternoon	10.79	0.47	15.56	0.72	11.88	0.52
RF	No W-No T	Night	11.90	0.48	10.87	0.48	8.43	0.34
SVR	All	Morning	12.97	0.47	25.91	0.95	21.10	0.82
SVR	All	Afternoon	13.33	0.53	19.32	0.81	14.75	0.62
SVR	All	Night	18.53	0.68	17.00	0.66	13.24	0.56
SVR	No W	Morning	13.28	0.48	26.45	0.96	21.57	0.83
SVR	No W	Afternoon	13.44	0.53	19.45	0.81	14.87	0.62
SVR	No W	Night	18.42	0.69	16.98	0.66	13.17	0.56
SVR	No T	Morning	12.95	0.47	25.87	0.95	21.07	0.82
SVR	No T	Afternoon	13.74	0.54	19.79	0.81	15.18	0.63
SVR	No T	Night	21.25	0.77	19.00	0.70	14.95	0.62
SVR	No W-No T	Morning	13.43	0.48	26.72	0.97	21.81	0.84
SVR	No W-No T	Afternoon	14.58	0.56	20.76	0.83	16.03	0.66
SVR	No W-No T	Night	24.76	0.85	21.26	0.73	17.04	0.67
FNN	All	Morning	13.21	0.48	26.26	0.96	21.47	0.83
FNN	All	Afternoon	12.95	0.52	18.89	0.80	14.36	0.61
FNN	All	Night	17.84	0.67	16.54	0.65	12.79	0.54
FNN	No W	Morning	9.53	0.40	19.44	0.78	15.52	0.65

FNN	No W	Afternoon	10.37	0.46	15.31	0.72	11.55	0.51
FNN	No W	Night	17.49	0.64	15.91	0.59	12.54	0.50
FNN	No T	Morning	13.09	0.47	25.90	0.98	21.08	0.82
FNN	No T	Afternoon	14.13	0.54	19.34	0.75	15.32	0.61
FNN	No T	Night	20.98	0.79	18.70	0.71	14.62	0.61
FNN	No W-No T	Morning	11.65	0.45	23.17	0.83	18.97	0.73
FNN	No W-No T	Afternoon	11.93	0.47	17.11	0.72	13.27	0.54
FNN	No W-No T	Night	17.49	0.64	16.05	0.62	12.56	0.51

**Method**	**Input variable**	**Risk group**	**SMAPE (mean)**	**SMAPE (st.dev.)**	**RMSE (mean)**	**RMSE (st.dev.)**	**MAE (mean)**	**MAE (st.dev.)**
SARIMA-1		Low	6.92	0.30	18.86	0.81	14.81	0.63
SARIMA-1		Medium	9.30	0.38	9.82	0.37	7.76	0.32
SARIMA-1		High	12.52	0.50	7.71	0.28	6.15	0.24
SARIMAX-1		Low	8.22	0.31	21.70	0.83	17.54	0.67
SARIMAX-1		Medium	10.25	0.42	10.87	0.40	8.58	0.35
SARIMAX-1		High	14.58	0.61	8.99	0.33	7.04	0.29
SARIMA-7		Low	7.40	0.32	20.27	0.85	15.85	0.67
SARIMA-7		Medium	9.28	0.38	9.86	0.38	7.74	0.32
SARIMA-7		High	12.68	0.50	7.78	0.28	6.23	0.24
SARIMAX-7		Low	13.50	0.64	45.32	4.10	30.06	1.76
SARIMAX-7		Medium	14.23	0.67	20.54	2.84	12.78	0.82
SARIMAX-7		High	23.04	0.99	20.29	2.51	12.26	0.83
SARIMA-14		Low	7.77	0.33	21.52	0.87	16.70	0.71
SARIMA-14		Medium	9.47	0.39	10.03	0.37	7.91	0.32
SARIMA-14		High	12.77	0.52	7.87	0.29	6.26	0.25
SARIMAX-14		Low	22.94	1.16	94.73	10.84	53.81	4.14
SARIMAX-14		Medium	19.62	1.12	39.32	7.86	19.23	1.88
SARIMAX-14		High	36.45	1.74	42.00	5.81	20.81	1.90
SARIMA-28		Low	9.40	0.38	25.64	0.97	20.26	0.82
SARIMA-28		Medium	9.67	0.39	10.20	0.37	8.08	0.32
SARIMA-28		High	13.23	0.53	8.18	0.31	6.50	0.26
SARIMAX-28		Low	47.65	2.10	207.45	22.98	114.69	9.12
SARIMAX-28		Medium	31.56	1.58	79.84	16.31	34.58	3.86
SARIMAX-28		High	70.90	3.04	91.91	12.29	43.06	4.25
RF	All	Low	8.59	0.35	23.38	0.97	18.25	0.77
RF	All	Medium	10.21	0.40	10.73	0.42	8.47	0.35
RF	All	High	14.31	0.55	8.82	0.32	7.00	0.29
RF	No W	Low	8.91	0.34	23.82	0.94	18.86	0.75
RF	No W	Medium	10.37	0.39	10.88	0.43	8.62	0.34
RF	No W	High	14.15	0.56	8.80	0.32	6.93	0.29
RF	No T	Low	9.95	0.37	26.19	0.93	20.94	0.81
RF	No T	Medium	10.96	0.41	11.33	0.42	9.07	0.35
RF	No T	High	13.35	0.52	8.22	0.30	6.54	0.26
RF	No W-No T	Low	9.06	0.35	24.41	0.98	19.26	0.77
RF	No W-No T	Medium	10.81	0.44	11.67	0.47	9.03	0.38
RF	No W-No T	High	14.65	0.55	8.88	0.32	7.15	0.28
SVR	All	Low	12.92	0.51	35.30	1.41	27.47	1.18
SVR	All	Medium	15.58	0.52	15.85	0.52	12.84	0.47
SVR	All	High	21.69	0.73	12.83	0.43	10.38	0.39
SVR	No W	Low	13.40	0.53	36.41	1.43	28.42	1.21
SVR	No W	Medium	16.11	0.54	16.30	0.53	13.24	0.48
SVR	No W	High	23.08	0.75	13.46	0.44	10.96	0.41

SVR	No T	Low	13.56	0.53	36.64	1.42	28.72	1.21
SVR	No T	Medium	16.11	0.53	16.29	0.53	13.24	0.48
SVR	No T	High	21.11	0.71	12.56	0.42	10.13	0.39
SVR	No W-No T	Low	15.40	0.56	40.40	1.47	32.25	1.28
SVR	No W-No T	Medium	16.92	0.55	16.94	0.54	13.84	0.50
SVR	No W-No T	High	20.99	0.72	12.53	0.42	10.09	0.39
FNN	All	Low	9.25	0.40	26.04	1.12	19.76	0.90
FNN	All	Medium	12.89	0.46	13.20	0.46	10.61	0.40
FNN	All	High	19.95	0.67	11.80	0.39	9.53	0.36
FNN	No W	Low	12.32	0.50	32.93	1.30	25.73	1.09
FNN	No W	Medium	14.16	0.46	13.95	0.47	11.55	0.40
FNN	No W	High	21.07	0.69	12.23	0.40	9.99	0.37
FNN	No T	Low	65.67	0.99	109.74	1.55	105.44	1.64
FNN	No T	Medium	67.31	0.89	43.78	0.56	42.24	0.59
FNN	No T	High	72.84	1.00	28.12	0.47	26.66	0.46
FNN	No W-No T	Low	10.73	0.46	29.76	1.25	23.06	1.04
FNN	No W-No T	Medium	13.24	0.44	13.56	0.46	11.02	0.40
FNN	No W-No T	High	18.80	0.68	11.53	0.40	9.14	0.37
