# Supplementary material for: Forecasting emergency department visits in the reference hospital of the Balearic Islands: The role of tourist and weather data
Source: PLoS One. 2026 Mar 13;21(3):e0343713. doi: 10.1371/journal.pone.0343713 (PMC12987453; doi:10.1371/journal.pone.0343713)
Supplement: S9 Table — For each pair of models (with the respective input variables) in the table, we report the fraction of bootstrap samples for which the two modes have equivalent predictive accuracy according to the Diebold-Mariano test. First table: shift-based predictions. Second table: risk-group-based predictions. (PDF) [file pone.0343713.s009.pdf]

| Method 1 | Input variable 1 | Method 2 | Input variable 2 | Shift | Fraction of equivalent samples |
|---|---|---|---|---|---|
| SARIMA-1 | | SARIMA-7 | | Morning | 0.988 |
| SARIMA-1 | | SARIMA-7 | | Afternoon | 0.924 |
| SARIMA-1 | | SARIMA-7 | | Night | 0.471 |
| SARIMA-1 | | SARIMA-14 | | Morning | 0.791 |
| SARIMA-1 | | SARIMA-14 | | Afternoon | 0.795 |
| SARIMA-1 | | SARIMA-14 | | Night | 0.035 |
| SARIMA-1 | | SARIMA-28 | | Morning | 0.267 |
| SARIMA-1 | | SARIMA-28 | | Afternoon | 0.127 |
| SARIMA-1 | | SARIMA-28 | | Night | 0.0 |
| SARIMA-7 | | SARIMA-14 | | Morning | 0.869 |
| SARIMA-7 | | SARIMA-14 | | Afternoon | 0.982 |
| SARIMA-7 | | SARIMA-14 | | Night | 0.643 |
| SARIMA-7 | | SARIMA-28 | | Morning | 0.296 |
| SARIMA-7 | | SARIMA-28 | | Afternoon | 0.359 |
| SARIMA-7 | | SARIMA-28 | | Night | 0.045 |
| SARIMA-14 | | SARIMA-28 | | Morning | 0.833 |
| SARIMA-14 | | SARIMA-28 | | Afternoon | 0.604 |
| SARIMA-14 | | SARIMA-28 | | Night | 0.443 |
| SARIMA-1 | | RF | No W | Morning | 0.583 |
| SARIMA-1 | | RF | No W | Afternoon | 0.826 |
| SARIMA-1 | | RF | No W | Night | 0.675 |
| SARIMA-1 | | RF | No W-No T | Morning | 0.245 |
| SARIMA-1 | | RF | No W-No T | Afternoon | 0.403 |
| SARIMA-1 | | RF | No W-No T | Night | 0.748 |
| SARIMA-7 | | RF | No W | Morning | 0.722 |
| SARIMA-7 | | RF | No W | Afternoon | 0.966 |
| SARIMA-7 | | RF | No W | Night | 0.996 |
| SARIMA-7 | | RF | No W-No T | Morning | 0.385 |
| SARIMA-7 | | RF | No W-No T | Afternoon | 0.736 |
| SARIMA-7 | | RF | No W-No T | Night | 0.998 |
| SARIMA-14 | | RF | No W | Morning | 0.897 |
| SARIMA-14 | | RF | No W | Afternoon | 0.996 |
| SARIMA-14 | | RF | No W | Night | 0.999 |
| SARIMA-14 | | RF | No W-No T | Morning | 0.614 |
| SARIMA-14 | | RF | No W-No T | Afternoon | 0.893 |
| SARIMA-14 | | RF | No W-No T | Night | 1.0 |
| SARIMA-28 | | RF | No W | Morning | 0.986 |
| SARIMA-28 | | RF | No W | Afternoon | 0.999 |
| SARIMA-28 | | RF | No W | Night | 0.924 |
| SARIMA-28 | | RF | No W-No T | Morning | 0.911 |
| SARIMA-28 | | RF | No W-No T | Afternoon | 1.0 |
| SARIMA-28 | | RF | No W-No T | Night | 0.809 |
| RF | All | RF | No T | Morning | 0.964 |
| RF | All | RF | No T | Afternoon | 0.349 |
| RF | All | RF | No T | Night | 0.065 |
| RF | All | RF | No W | Morning | 0.981 |
| RF | All | RF | No W | Afternoon | 0.954 |
| RF | All | RF | No W | Night | 0.962 |
| RF | All | RF | No W-No T | Morning | 0.997 |
| RF | All | RF | No W-No T | Afternoon | 0.568 |
| RF | All | RF | No W-No T | Night | 0.999 |
| RF | No T | RF | No W | Morning | 0.876 |

| | | | | | |
|---|---|---|---|---|---|
| RF | No T | RF | No W | Afternoon | 0.949 |
| RF | No T | RF | No W | Night | 0.453 |
| RF | No T | RF | No W-No T | Morning | 1.0 |
| RF | No T | RF | No W-No T | Afternoon | 1.0 |
| RF | No T | RF | No W-No T | Night | 0.255 |
| RF | No W | RF | No W-No T | Morning | 0.959 |
| RF | No W | RF | No W-No T | Afternoon | 0.893 |
| RF | No W | RF | No W-No T | Night | 0.996 |
| RF | All | SVR | All | Morning | 0.0 |
| RF | All | SVR | All | Afternoon | 0.0 |
| RF | All | SVR | All | Night | 0.0 |
| RF | All | SVR | No T | Morning | 0.0 |
| RF | All | SVR | No T | Afternoon | 0.0 |
| RF | All | SVR | No T | Night | 0.0 |
| RF | No T | SVR | All | Morning | 0.0 |
| RF | No T | SVR | All | Afternoon | 0.002 |
| RF | No T | SVR | All | Night | 0.0 |
| RF | All | SVR | No W | Morning | 0.0 |
| RF | All | SVR | No W | Afternoon | 0.0 |
| RF | All | SVR | No W | Night | 0.0 |
| RF | No W | SVR | All | Morning | 0.0 |
| RF | No W | SVR | All | Afternoon | 0.0 |
| RF | No W | SVR | All | Night | 0.0 |
| RF | All | SVR | No W-No T | Morning | 0.0 |
| RF | All | SVR | No W-No T | Afternoon | 0.0 |
| RF | All | SVR | No W-No T | Night | 0.0 |
| RF | No W-No T | SVR | All | Morning | 0.0 |
| RF | No W-No T | SVR | All | Afternoon | 0.005 |
| RF | No W-No T | SVR | All | Night | 0.0 |
| RF | No T | SVR | No T | Morning | 0.0 |
| RF | No T | SVR | No T | Afternoon | 0.0 |
| RF | No T | SVR | No T | Night | 0.0 |
| RF | No T | SVR | No W | Morning | 0.0 |
| RF | No T | SVR | No W | Afternoon | 0.0 |
| RF | No T | SVR | No W | Night | 0.001 |
| RF | No W | SVR | No T | Morning | 0.0 |
| RF | No W | SVR | No T | Afternoon | 0.0 |
| RF | No W | SVR | No T | Night | 0.0 |
| RF | No T | SVR | No W-No T | Morning | 0.0 |
| RF | No T | SVR | No W-No T | Afternoon | 0.0 |
| RF | No T | SVR | No W-No T | Night | 0.0 |
| RF | No W-No T | SVR | No T | Morning | 0.0 |
| RF | No W-No T | SVR | No T | Afternoon | 0.001 |
| RF | No W-No T | SVR | No T | Night | 0.0 |
| RF | No W | SVR | No W | Morning | 0.0 |
| RF | No W | SVR | No W | Afternoon | 0.0 |
| RF | No W | SVR | No W | Night | 0.0 |
| RF | No W | SVR | No W-No T | Morning | 0.0 |
| RF | No W | SVR | No W-No T | Afternoon | 0.0 |
| RF | No W | SVR | No W-No T | Night | 0.0 |
| RF | No W-No T | SVR | No W | Morning | 0.0 |
| RF | No W-No T | SVR | No W | Afternoon | 0.004 |
| RF | No W-No T | SVR | No W | Night | 0.0 |
| RF | No W-No T | SVR | No W-No T | Morning | 0.0 |

| | | | | | |
|---|---|---|---|---|---|
| RF | No W-No T | SVR | No W-No T | Afternoon | 0.0 |
| RF | No W-No T | SVR | No W-No T | Night | 0.0 |
| RF | All | FNN | All | Morning | 0.0 |
| RF | All | FNN | All | Afternoon | 0.0 |
| RF | All | FNN | All | Night | 0.0 |
| RF | All | FNN | No T | Morning | 0.0 |
| RF | All | FNN | No T | Afternoon | 0.0 |
| RF | All | FNN | No T | Night | 0.0 |
| RF | No T | FNN | All | Morning | 0.0 |
| RF | No T | FNN | All | Afternoon | 0.041 |
| RF | No T | FNN | All | Night | 0.006 |
| RF | All | FNN | No W | Morning | 0.999 |
| RF | All | FNN | No W | Afternoon | 0.993 |
| RF | All | FNN | No W | Night | 0.0 |
| RF | No W | FNN | All | Morning | 0.0 |
| RF | No W | FNN | All | Afternoon | 0.004 |
| RF | No W | FNN | All | Night | 0.0 |
| RF | All | FNN | No W-No T | Morning | 0.012 |
| RF | All | FNN | No W-No T | Afternoon | 0.093 |
| RF | All | FNN | No W-No T | Night | 0.0 |
| RF | No W-No T | FNN | All | Morning | 0.0 |
| RF | No W-No T | FNN | All | Afternoon | 0.072 |
| RF | No W-No T | FNN | All | Night | 0.0 |
| RF | No T | FNN | No T | Morning | 0.0 |
| RF | No T | FNN | No T | Afternoon | 0.0 |
| RF | No T | FNN | No T | Night | 0.0 |
| RF | No T | FNN | No W | Morning | 1.0 |
| RF | No T | FNN | No W | Afternoon | 0.994 |
| RF | No T | FNN | No W | Night | 0.055 |
| RF | No W | FNN | No T | Morning | 0.0 |
| RF | No W | FNN | No T | Afternoon | 0.0 |
| RF | No W | FNN | No T | Night | 0.0 |
| RF | No T | FNN | No W-No T | Morning | 0.104 |
| RF | No T | FNN | No W-No T | Afternoon | 0.756 |
| RF | No T | FNN | No W-No T | Night | 0.024 |
| RF | No W-No T | FNN | No T | Morning | 0.0 |
| RF | No W-No T | FNN | No T | Afternoon | 0.0 |
| RF | No W-No T | FNN | No T | Night | 0.0 |
| RF | No W | FNN | No W | Morning | 0.988 |
| RF | No W | FNN | No W | Afternoon | 1.0 |
| RF | No W | FNN | No W | Night | 0.0 |
| RF | No W | FNN | No W-No T | Morning | 0.003 |
| RF | No W | FNN | No W-No T | Afternoon | 0.365 |
| RF | No W | FNN | No W-No T | Night | 0.0 |
| RF | No W-No T | FNN | No W | Morning | 1.0 |
| RF | No W-No T | FNN | No W | Afternoon | 0.994 |
| RF | No W-No T | FNN | No W | Night | 0.0 |
| RF | No W-No T | FNN | No W-No T | Morning | 0.048 |
| RF | No W-No T | FNN | No W-No T | Afternoon | 0.748 |
| RF | No W-No T | FNN | No W-No T | Night | 0.0 |
| SVR | All | SVR | No T | Morning | 0.83 |
| SVR | All | SVR | No T | Afternoon | 0.0 |
| SVR | All | SVR | No T | Night | 0.0 |
| SVR | All | SVR | No W | Morning | 0.0 |

| | | | | | |
|---|---|---|---|---|---|
| SVR | All | SVR | No W | Afternoon | 0.035 |
| SVR | All | SVR | No W | Night | 0.925 |
| SVR | All | SVR | No W-No T | Morning | 0.0 |
| SVR | All | SVR | No W-No T | Afternoon | 0.0 |
| SVR | All | SVR | No W-No T | Night | 0.0 |
| SVR | No T | SVR | No W | Morning | 0.0 |
| SVR | No T | SVR | No W | Afternoon | 0.003 |
| SVR | No T | SVR | No W | Night | 0.0 |
| SVR | No T | SVR | No W-No T | Morning | 0.0 |
| SVR | No T | SVR | No W-No T | Afternoon | 0.0 |
| SVR | No T | SVR | No W-No T | Night | 0.0 |
| SVR | No W | SVR | No W-No T | Morning | 0.0 |
| SVR | No W | SVR | No W-No T | Afternoon | 0.0 |
| SVR | No W | SVR | No W-No T | Night | 0.0 |
| SVR | All | FNN | All | Morning | 0.619 |
| SVR | All | FNN | All | Afternoon | 0.398 |
| SVR | All | FNN | All | Night | 0.061 |
| SVR | All | FNN | No T | Morning | 1.0 |
| SVR | All | FNN | No T | Afternoon | 0.965 |
| SVR | All | FNN | No T | Night | 0.011 |
| SVR | No T | FNN | All | Morning | 0.383 |
| SVR | No T | FNN | All | Afternoon | 0.007 |
| SVR | No T | FNN | All | Night | 0.0 |
| SVR | All | FNN | No W | Morning | 0.0 |
| SVR | All | FNN | No W | Afternoon | 0.0 |
| SVR | All | FNN | No W | Night | 0.86 |
| SVR | No W | FNN | All | Morning | 0.998 |
| SVR | No W | FNN | All | Afternoon | 0.021 |
| SVR | No W | FNN | All | Night | 0.009 |
| SVR | All | FNN | No W-No T | Morning | 0.112 |
| SVR | All | FNN | No W-No T | Afternoon | 0.188 |
| SVR | All | FNN | No W-No T | Night | 0.576 |
| SVR | No W-No T | FNN | All | Morning | 0.855 |
| SVR | No W-No T | FNN | All | Afternoon | 0.0 |
| SVR | No W-No T | FNN | All | Night | 0.0 |
| SVR | No T | FNN | No T | Morning | 1.0 |
| SVR | No T | FNN | No T | Afternoon | 1.0 |
| SVR | No T | FNN | No T | Night | 1.0 |
| SVR | No T | FNN | No W | Morning | 0.0 |
| SVR | No T | FNN | No W | Afternoon | 0.0 |
| SVR | No T | FNN | No W | Night | 0.002 |
| SVR | No W | FNN | No T | Morning | 0.998 |
| SVR | No W | FNN | No T | Afternoon | 0.988 |
| SVR | No W | FNN | No T | Night | 0.006 |
| SVR | No T | FNN | No W-No T | Morning | 0.11 |
| SVR | No T | FNN | No W-No T | Afternoon | 0.036 |
| SVR | No T | FNN | No W-No T | Night | 0.0 |
| SVR | No W-No T | FNN | No T | Morning | 0.993 |
| SVR | No W-No T | FNN | No T | Afternoon | 0.997 |
| SVR | No W-No T | FNN | No T | Night | 0.001 |
| SVR | No W | FNN | No W | Morning | 0.0 |
| SVR | No W | FNN | No W | Afternoon | 0.0 |
| SVR | No W | FNN | No W | Night | 0.892 |
| SVR | No W | FNN | No W-No T | Morning | 0.014 |

| | | | | | |
|---|---|---|---|---|---|
| SVR | No W | FNN | No W-No T | Afternoon | 0.101 |
| SVR | No W | FNN | No W-No T | Night | 0.599 |
| SVR | No W-No T | FNN | No W | Morning | 0.0 |
| SVR | No W-No T | FNN | No W | Afternoon | 0.0 |
| SVR | No W-No T | FNN | No W | Night | 0.0 |
| SVR | No W-No T | FNN | No W-No T | Morning | 0.007 |
| SVR | No W-No T | FNN | No W-No T | Afternoon | 0.0 |
| SVR | No W-No T | FNN | No W-No T | Night | 0.0 |
| FNN | All | FNN | No T | Morning | 1.0 |
| FNN | All | FNN | No T | Afternoon | 0.817 |
| FNN | All | FNN | No T | Night | 0.0 |
| FNN | All | FNN | No W | Morning | 0.0 |
| FNN | All | FNN | No W | Afternoon | 0.0 |
| FNN | All | FNN | No W | Night | 0.997 |
| FNN | All | FNN | No W-No T | Morning | 0.004 |
| FNN | All | FNN | No W-No T | Afternoon | 0.379 |
| FNN | All | FNN | No W-No T | Night | 0.987 |
| FNN | No T | FNN | No W | Morning | 0.0 |
| FNN | No T | FNN | No W | Afternoon | 0.0 |
| FNN | No T | FNN | No W | Night | 0.0 |
| FNN | No T | FNN | No W-No T | Morning | 0.749 |
| FNN | No T | FNN | No W-No T | Afternoon | 0.217 |
| FNN | No T | FNN | No W-No T | Night | 0.0 |
| FNN | No W | FNN | No W-No T | Morning | 0.0 |
| FNN | No W | FNN | No W-No T | Afternoon | 0.108 |
| FNN | No W | FNN | No W-No T | Night | 1.0 |

| **Method 1** | **Input variable 1** | **Method 2** | **Input variable 2** | **Risk group** | **Fraction of equivalent samples** |
|---|---|---|---|---|---|
| SARIMA-1 | | SARIMA-7 | | Low | 0.817 |
| SARIMA-1 | | SARIMA-7 | | Medium | 1.0 |
| SARIMA-1 | | SARIMA-7 | | High | 0.997 |
| SARIMA-1 | | SARIMA-14 | | Low | 0.518 |
| SARIMA-1 | | SARIMA-14 | | Medium | 0.995 |
| SARIMA-1 | | SARIMA-14 | | High | 0.989 |
| SARIMA-1 | | SARIMA-28 | | Low | 0.0 |
| SARIMA-1 | | SARIMA-28 | | Medium | 0.963 |
| SARIMA-1 | | SARIMA-28 | | High | 0.902 |
| SARIMA-7 | | SARIMA-14 | | Low | 0.938 |
| SARIMA-7 | | SARIMA-14 | | Medium | 0.97 |
| SARIMA-7 | | SARIMA-14 | | High | 0.998 |
| SARIMA-7 | | SARIMA-28 | | Low | 0.0 |
| SARIMA-7 | | SARIMA-28 | | Medium | 0.935 |
| SARIMA-7 | | SARIMA-28 | | High | 0.95 |
| SARIMA-14 | | SARIMA-28 | | Low | 0.0 |
| SARIMA-14 | | SARIMA-28 | | Medium | 0.991 |
| SARIMA-14 | | SARIMA-28 | | High | 0.959 |
| SARIMA-1 | | RF | No W | Low | 0.062 |
| SARIMA-1 | | RF | No W | Medium | 0.796 |
| SARIMA-1 | | RF | No W | High | 0.747 |
| SARIMA-1 | | RF | No W-No T | Low | 0.029 |
| SARIMA-1 | | RF | No W-No T | Medium | 0.495 |
| SARIMA-1 | | RF | No W-No T | High | 0.384 |

| | | | | | |
|---|---|---|---|---|---|
| SARIMA-7 | | RF | No W | Low | 0.416 |
| SARIMA-7 | | RF | No W | Medium | 0.787 |
| SARIMA-7 | | RF | No W | High | 0.824 |
| SARIMA-7 | | RF | No W-No T | Low | 0.258 |
| SARIMA-7 | | RF | No W-No T | Medium | 0.474 |
| SARIMA-7 | | RF | No W-No T | High | 0.51 |
| SARIMA-14 | | RF | No W | Low | 0.798 |
| SARIMA-14 | | RF | No W | Medium | 0.91 |
| SARIMA-14 | | RF | No W | High | 0.869 |
| SARIMA-14 | | RF | No W-No T | Low | 0.646 |
| SARIMA-14 | | RF | No W-No T | Medium | 0.63 |
| SARIMA-14 | | RF | No W-No T | High | 0.615 |
| SARIMA-28 | | RF | No W | Low | 0.996 |
| SARIMA-28 | | RF | No W | Medium | 0.97 |
| SARIMA-28 | | RF | No W | High | 0.979 |
| SARIMA-28 | | RF | No W-No T | Low | 1.0 |
| SARIMA-28 | | RF | No W-No T | Medium | 0.789 |
| SARIMA-28 | | RF | No W-No T | High | 0.905 |
| RF | All | RF | No T | Low | 0.005 |
| RF | All | RF | No T | Medium | 0.192 |
| RF | All | RF | No T | High | 0.404 |
| RF | All | RF | No W | Low | 0.813 |
| RF | All | RF | No W | Medium | 0.999 |
| RF | All | RF | No W | High | 1.0 |
| RF | All | RF | No W-No T | Low | 0.935 |
| RF | All | RF | No W-No T | Medium | 0.948 |
| RF | All | RF | No W-No T | High | 0.995 |
| RF | No T | RF | No W | Low | 0.349 |
| RF | No T | RF | No W | Medium | 0.889 |
| RF | No T | RF | No W | High | 0.871 |
| RF | No T | RF | No W-No T | Low | 0.459 |
| RF | No T | RF | No W-No T | Medium | 0.999 |
| RF | No T | RF | No W-No T | High | 0.375 |
| RF | No W | RF | No W-No T | Low | 0.998 |
| RF | No W | RF | No W-No T | Medium | 0.985 |
| RF | No W | RF | No W-No T | High | 0.959 |
| RF | All | SVR | All | Low | 0.0 |
| RF | All | SVR | All | Medium | 0.0 |
| RF | All | SVR | All | High | 0.0 |
| RF | All | SVR | No T | Low | 0.0 |
| RF | All | SVR | No T | Medium | 0.0 |
| RF | All | SVR | No T | High | 0.0 |
| RF | No T | SVR | All | Low | 0.002 |
| RF | No T | SVR | All | Medium | 0.0 |
| RF | No T | SVR | All | High | 0.0 |
| RF | All | SVR | No W | Low | 0.0 |
| RF | All | SVR | No W | Medium | 0.0 |
| RF | All | SVR | No W | High | 0.0 |
| RF | No W | SVR | All | Low | 0.0 |
| RF | No W | SVR | All | Medium | 0.0 |
| RF | No W | SVR | All | High | 0.0 |
| RF | All | SVR | No W-No T | Low | 0.0 |
| RF | All | SVR | No W-No T | Medium | 0.0 |
| RF | All | SVR | No W-No T | High | 0.0 |

| | | | | | |
|---|---|---|---|---|---|
| RF | No W-No T | SVR | All | Low | 0.0 |
| RF | No W-No T | SVR | All | Medium | 0.0 |
| RF | No W-No T | SVR | All | High | 0.0 |
| RF | No T | SVR | No T | Low | 0.0 |
| RF | No T | SVR | No T | Medium | 0.0 |
| RF | No T | SVR | No T | High | 0.0 |
| RF | No T | SVR | No W | Low | 0.0 |
| RF | No T | SVR | No W | Medium | 0.0 |
| RF | No T | SVR | No W | High | 0.0 |
| RF | No W | SVR | No T | Low | 0.0 |
| RF | No W | SVR | No T | Medium | 0.0 |
| RF | No W | SVR | No T | High | 0.0 |
| RF | No T | SVR | No W-No T | Low | 0.0 |
| RF | No T | SVR | No W-No T | Medium | 0.0 |
| RF | No T | SVR | No W-No T | High | 0.0 |
| RF | No W-No T | SVR | No T | Low | 0.0 |
| RF | No W-No T | SVR | No T | Medium | 0.0 |
| RF | No W-No T | SVR | No T | High | 0.0 |
| RF | No W | SVR | No W | Low | 0.0 |
| RF | No W | SVR | No W | Medium | 0.0 |
| RF | No W | SVR | No W | High | 0.0 |
| RF | No W | SVR | No W-No T | Low | 0.0 |
| RF | No W | SVR | No W-No T | Medium | 0.0 |
| RF | No W | SVR | No W-No T | High | 0.0 |
| RF | No W-No T | SVR | No W | Low | 0.0 |
| RF | No W-No T | SVR | No W | Medium | 0.0 |
| RF | No W-No T | SVR | No W | High | 0.0 |
| RF | No W-No T | SVR | No W-No T | Low | 0.0 |
| RF | No W-No T | SVR | No W-No T | Medium | 0.0 |
| RF | No W-No T | SVR | No W-No T | High | 0.0 |
| RF | All | FNN | All | Low | 0.936 |
| RF | All | FNN | All | Medium | 0.001 |
| RF | All | FNN | All | High | 0.0 |
| RF | All | FNN | No T | Low | 0.0 |
| RF | All | FNN | No T | Medium | 0.0 |
| RF | All | FNN | No T | High | 0.0 |
| RF | No T | FNN | All | Low | 0.973 |
| RF | No T | FNN | All | Medium | 0.222 |
| RF | No T | FNN | All | High | 0.0 |
| RF | All | FNN | No W | Low | 0.0 |
| RF | All | FNN | No W | Medium | 0.0 |
| RF | All | FNN | No W | High | 0.0 |
| RF | No W | FNN | All | Low | 0.996 |
| RF | No W | FNN | All | Medium | 0.015 |
| RF | No W | FNN | All | High | 0.0 |
| RF | All | FNN | No W-No T | Low | 0.062 |
| RF | All | FNN | No W-No T | Medium | 0.0 |
| RF | All | FNN | No W-No T | High | 0.0 |
| RF | No W-No T | FNN | All | Low | 1.0 |
| RF | No W-No T | FNN | All | Medium | 0.382 |
| RF | No W-No T | FNN | All | High | 0.0 |
| RF | No T | FNN | No T | Low | 0.0 |
| RF | No T | FNN | No T | Medium | 0.0 |
| RF | No T | FNN | No T | High | 0.0 |

| | | | | | |
|---|---|---|---|---|---|
| RF | No T | FNN | No W | Low | 0.043 |
| RF | No T | FNN | No W | Medium | 0.0 |
| RF | No T | FNN | No W | High | 0.0 |
| RF | No W | FNN | No T | Low | 0.0 |
| RF | No W | FNN | No T | Medium | 0.0 |
| RF | No W | FNN | No T | High | 0.0 |
| RF | No T | FNN | No W-No T | Low | 0.97 |
| RF | No T | FNN | No W-No T | Medium | 0.035 |
| RF | No T | FNN | No W-No T | High | 0.0 |
| RF | No W-No T | FNN | No T | Low | 0.0 |
| RF | No W-No T | FNN | No T | Medium | 0.0 |
| RF | No W-No T | FNN | No T | High | 0.0 |
| RF | No W | FNN | No W | Low | 0.0 |
| RF | No W | FNN | No W | Medium | 0.0 |
| RF | No W | FNN | No W | High | 0.0 |
| RF | No W | FNN | No W-No T | Low | 0.244 |
| RF | No W | FNN | No W-No T | Medium | 0.0 |
| RF | No W | FNN | No W-No T | High | 0.0 |
| RF | No W-No T | FNN | No W | Low | 0.0 |
| RF | No W-No T | FNN | No W | Medium | 0.001 |
| RF | No W-No T | FNN | No W | High | 0.0 |
| RF | No W-No T | FNN | No W-No T | Low | 0.207 |
| RF | No W-No T | FNN | No W-No T | Medium | 0.044 |
| RF | No W-No T | FNN | No W-No T | High | 0.006 |
| SVR | All | SVR | No T | Low | 0.0 |
| SVR | All | SVR | No T | Medium | 0.0 |
| SVR | All | SVR | No T | High | 0.0 |
| SVR | All | SVR | No W | Low | 0.0 |
| SVR | All | SVR | No W | Medium | 0.0 |
| SVR | All | SVR | No W | High | 0.0 |
| SVR | All | SVR | No W-No T | Low | 0.0 |
| SVR | All | SVR | No W-No T | Medium | 0.0 |
| SVR | All | SVR | No W-No T | High | 0.0 |
| SVR | No T | SVR | No W | Low | 0.01 |
| SVR | No T | SVR | No W | Medium | 1.0 |
| SVR | No T | SVR | No W | High | 0.0 |
| SVR | No T | SVR | No W-No T | Low | 0.0 |
| SVR | No T | SVR | No W-No T | Medium | 0.0 |
| SVR | No T | SVR | No W-No T | High | 0.977 |
| SVR | No W | SVR | No W-No T | Low | 0.0 |
| SVR | No W | SVR | No W-No T | Medium | 0.0 |
| SVR | No W | SVR | No W-No T | High | 0.0 |
| SVR | All | FNN | All | Low | 0.0 |
| SVR | All | FNN | All | Medium | 0.0 |
| SVR | All | FNN | All | High | 0.4 |
| SVR | All | FNN | No T | Low | 0.0 |
| SVR | All | FNN | No T | Medium | 0.0 |
| SVR | All | FNN | No T | High | 0.0 |
| SVR | No T | FNN | All | Low | 0.0 |
| SVR | No T | FNN | All | Medium | 0.0 |
| SVR | No T | FNN | All | High | 0.835 |
| SVR | All | FNN | No W | Low | 0.986 |
| SVR | All | FNN | No W | Medium | 0.599 |
| SVR | All | FNN | No W | High | 0.989 |

| | | | | | |
|---|---|---|---|---|---|
| SVR | No W | FNN | All | Low | 0.0 |
| SVR | No W | FNN | All | Medium | 0.0 |
| SVR | No W | FNN | All | High | 0.0 |
| SVR | All | FNN | No W-No T | Low | 0.0 |
| SVR | All | FNN | No W-No T | Medium | 0.0 |
| SVR | All | FNN | No W-No T | High | 0.0 |
| SVR | No W-No T | FNN | All | Low | 0.0 |
| SVR | No W-No T | FNN | All | Medium | 0.0 |
| SVR | No W-No T | FNN | All | High | 0.884 |
| SVR | No T | FNN | No T | Low | 0.0 |
| SVR | No T | FNN | No T | Medium | 0.0 |
| SVR | No T | FNN | No T | High | 0.0 |
| SVR | No T | FNN | No W | Low | 0.826 |
| SVR | No T | FNN | No W | Medium | 0.155 |
| SVR | No T | FNN | No W | High | 0.999 |
| SVR | No W | FNN | No T | Low | 0.0 |
| SVR | No W | FNN | No T | Medium | 0.0 |
| SVR | No W | FNN | No T | High | 0.0 |
| SVR | No T | FNN | No W-No T | Low | 0.0 |
| SVR | No T | FNN | No W-No T | Medium | 0.0 |
| SVR | No T | FNN | No W-No T | High | 0.0 |
| SVR | No W-No T | FNN | No T | Low | 0.0 |
| SVR | No W-No T | FNN | No T | Medium | 0.0 |
| SVR | No W-No T | FNN | No T | High | 0.0 |
| SVR | No W | FNN | No W | Low | 0.914 |
| SVR | No W | FNN | No W | Medium | 0.2 |
| SVR | No W | FNN | No W | High | 0.202 |
| SVR | No W | FNN | No W-No T | Low | 0.0 |
| SVR | No W | FNN | No W-No T | Medium | 0.0 |
| SVR | No W | FNN | No W-No T | High | 0.0 |
| SVR | No W-No T | FNN | No W | Low | 0.0 |
| SVR | No W-No T | FNN | No W | Medium | 0.001 |
| SVR | No W-No T | FNN | No W | High | 0.999 |
| SVR | No W-No T | FNN | No W-No T | Low | 0.0 |
| SVR | No W-No T | FNN | No W-No T | Medium | 0.0 |
| SVR | No W-No T | FNN | No W-No T | High | 0.0 |
| FNN | All | FNN | No T | Low | 0.0 |
| FNN | All | FNN | No T | Medium | 0.0 |
| FNN | All | FNN | No T | High | 0.0 |
| FNN | All | FNN | No W | Low | 0.0 |
| FNN | All | FNN | No W | Medium | 0.762 |
| FNN | All | FNN | No W | High | 0.899 |
| FNN | All | FNN | No W-No T | Low | 0.398 |
| FNN | All | FNN | No W-No T | Medium | 0.999 |
| FNN | All | FNN | No W-No T | High | 0.828 |
| FNN | No T | FNN | No W | Low | 0.0 |
| FNN | No T | FNN | No W | Medium | 0.0 |
| FNN | No T | FNN | No W | High | 0.0 |
| FNN | No T | FNN | No W-No T | Low | 0.0 |
| FNN | No T | FNN | No W-No T | Medium | 0.0 |
| FNN | No T | FNN | No W-No T | High | 0.0 |
| FNN | No W | FNN | No W-No T | Low | 0.468 |
| FNN | No W | FNN | No W-No T | Medium | 0.927 |
| FNN | No W | FNN | No W-No T | High | 0.061 |
