# Supplementary material for: Forecasting emergency department visits in the reference hospital of the Balearic Islands: The role of tourist and weather data
Source: PLoS One. 2026 Mar 13;21(3):e0343713. doi: 10.1371/journal.pone.0343713 (PMC12987453; doi:10.1371/journal.pone.0343713)
Supplement: S10 Table — Model performance on the post-COVID dataset. The model tested is the RF model for all four input combinations. First table: shift-based predictions. Second table: risk-group-based predictions. (PDF) [file pone.0343713.s010.pdf]

Method	Input variables	Shift	SMAPE (mean)	SMAPE (st.dev.)	RMSE (mean)	RMSE (st.dev.)	MAE (mean)	MAE (st.dev.)
RF	All	Morning	15.19	0.72	33.45	1.56	24.88	1.18
RF	All	Afternoon	15.42	0.69	23.88	1.40	17.61	0.86
RF	All	Night	14.76	0.75	13.35	0.66	10.28	0.45
RF	No W	Morning	14.86	0.69	32.64	1.51	24.47	1.14
RF	No W	Afternoon	15.82	0.70	24.21	1.40	17.99	0.87
RF	No W	Night	15.31	0.74	13.60	0.64	10.63	0.44
RF	No T	Morning	14.96	0.71	33.07	1.52	24.54	1.16
RF	No T	Afternoon	16.25	0.72	24.76	1.43	18.39	0.89
RF	No T	Night	15.79	0.77	14.21	0.70	10.96	0.47
RF	No W-No T	Morning	15.16	0.70	33.32	1.51	24.94	1.15
RF	No W-No T	Afternoon	16.13	0.77	24.98	1.47	18.26	0.93
RF	No W-No T	Night	14.70	0.74	13.14	0.73	10.32	0.44

Method	Input variables	Risk group	SMAPE (mean)	SMAPE (st.dev.)	RMSE (mean)	RMSE (st.dev.)	MAE (mean)	MAE (st.dev.)
RF	All	Low	11.63	0.48	32.60	1.39	25.12	1.10
RF	All	Medium	15.73	0.73	18.38	0.94	13.37	0.66
RF	All	High	20.47	0.80	13.09	0.56	10.25	0.43
RF	No W	Low	12.25	0.50	33.99	1.43	26.37	1.13
RF	No W	Medium	15.27	0.71	17.91	0.92	13.04	0.64
RF	No W	High	19.85	0.78	12.74	0.53	10.00	0.42
RF	No T	Low	12.61	0.50	34.37	1.34	27.00	1.12
RF	No T	Medium	15.54	0.72	18.09	0.91	13.20	0.65
RF	No T	High	20.41	0.80	13.09	0.57	10.22	0.42
RF	No W-No T	Low	12.48	0.50	34.38	1.44	26.86	1.12
RF	No W-No T	Medium	15.87	0.70	18.33	0.88	13.61	0.63
RF	No W-No T	High	19.86	0.79	12.78	0.54	9.99	0.42
